# A three-headed plantaris muscle: evidence that the plantaris is not a vestigial muscle?

**DOI:** 10.1007/s00276-020-02478-8

**Published:** 2020-05-08

**Authors:** Łukasz Olewnik, N. Zielinska, P. Karauda, R. Shane Tubbs, M. Polguj

**Affiliations:** 1 Department of Anatomical Dissection and Donation, Medical University of Lodz, Lodz, Poland; 2 Department of Normal and Clinical Anatomy, Medical University of Lodz, Lodz, Poland; 3 Department of Neurosurgery, Tulane University School of Medicine, New Orleans, LA USA; 4 Department of Neurosurgery and Ochsner Neuroscience Institute, Ochsner Health System, New Orleans, LA USA; 5 Department of Anatomical Sciences, St. George’s University, St. George’s, Grenada

**Keywords:** Plantaris tendon, Plantaris muscle, Anatomical variations, Plantaris

## Abstract

The plantaris is a small muscle that typically originates at the lateral supracondylar line of the femur and the knee joint capsule, from where it continues distally, forming a long and slender tendon. However, considerable controversy surrounds the status of this seemingly inconspicuous muscle: is it a residual muscle, or one that it is just developing? In addition, both the proximal and distal attachments are highly morphologically variable. These variations can lead to many diseases. Interestingly, the course of the PM tendon is also variable. The present case study presents a new description of a complex origin type and a rare course of the PM tendon. Understanding of the PM and its tendon has clear clinical value and is a significant indicator of the development of interest in this overlooked muscle.

## Introduction

The plantaris muscle (PM) is typically described as small, short and fusiform. Together with the gastrocnemius (GM) and soleus (SM) muscles, it belongs to the superficial posterior compartment of the lower limb [22–24]. In most cases, the PM originates from the lateral supracondylar line of the femur and the knee joint capsule [15]. From this origin, the short muscle belly develops into a long, thin tendon, which usually runs in the “space” between the GM and the SM, inserting on to the medial calcaneus and adjacent fibrous tissues [7, 11, 22, 23, 31].

Morphological variations have been described more often in the distal attachment than the proximal attachment; however, both are characterized by frequent variation [7, 9, 11, 13, 17, 22, 23, 25].

The PM can be absent [10, 22, 23, 28], double [26] or bifurcated [14, 30], or it can follow an unusual course in relation to the neuromuscular bundle [24]. Therefore, it could be the currently most “spectacular” muscle, embroiled in controversy as to whether it is a vestigial muscle or one that is just developing?

The present case report describes a very rare example of a three-headed PM, and reveals a new variant of the course of the PM tendon relative to the Achilles tendon.

## Case report

### Variability regarding the number of muscle bellies

A 73-year-old female cadaver was subjected to routine anatomical dissection for research and teaching at the Department of Anatomical Dissection and Donation, Chair Anatomy and Histology, Medical University of Lodz. The right lower limb (knee and crural region) was dissected using standard techniques according to a specified protocol [19–21].

**Fig. 1:**
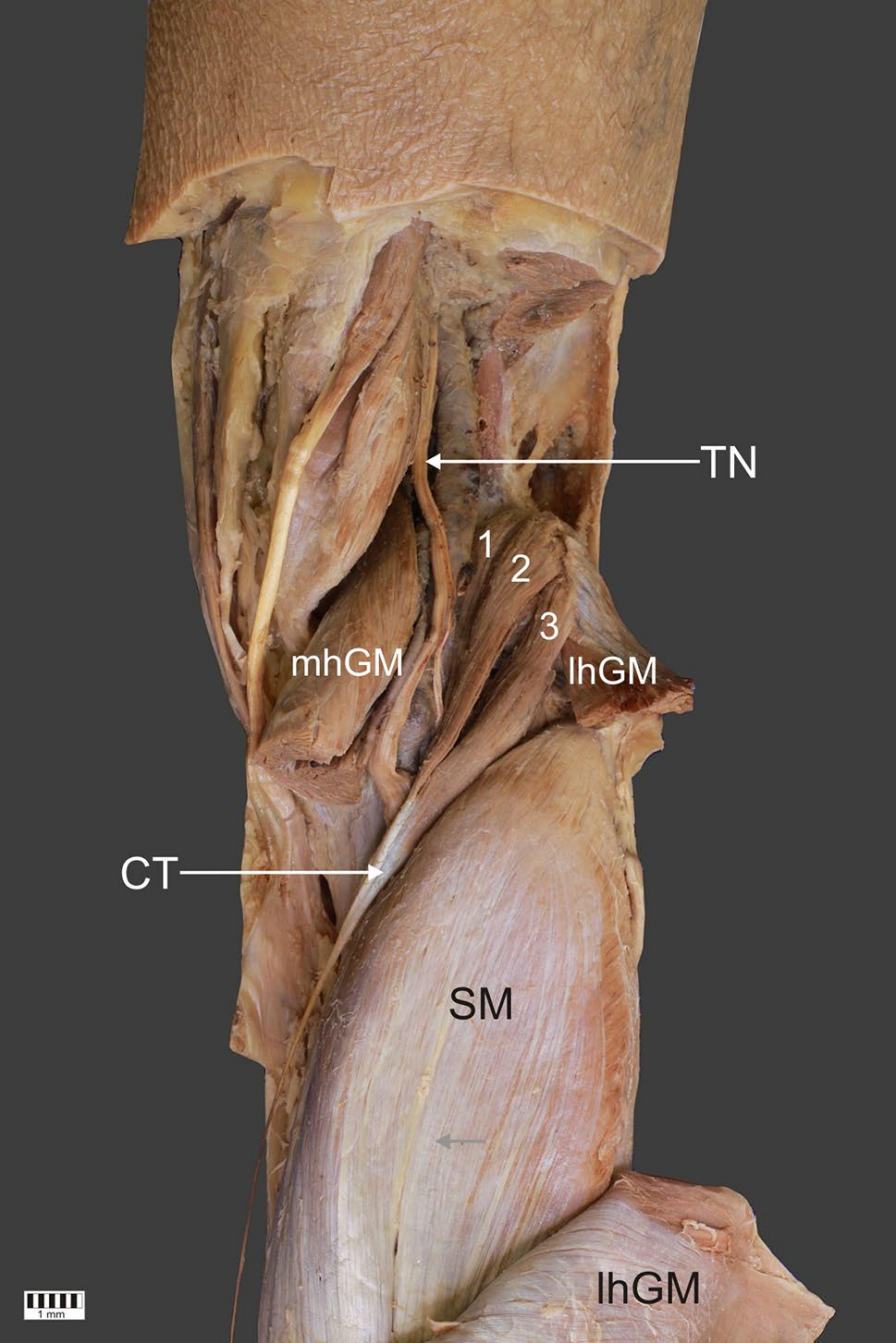
A rare three-headed plantaris muscle. *1* first head of the plantaris muscle, *2* s head of the plantaris muscle, *3* third head of the plantaris muscle, *TN* tibial nerve, *lhGM* lateral head of the gastrocnemius muscle, *mhGM* medial head of the gastrocnemius muscle, *CT* common tendon, *SM* soleus muscle

**Fig. 2:**
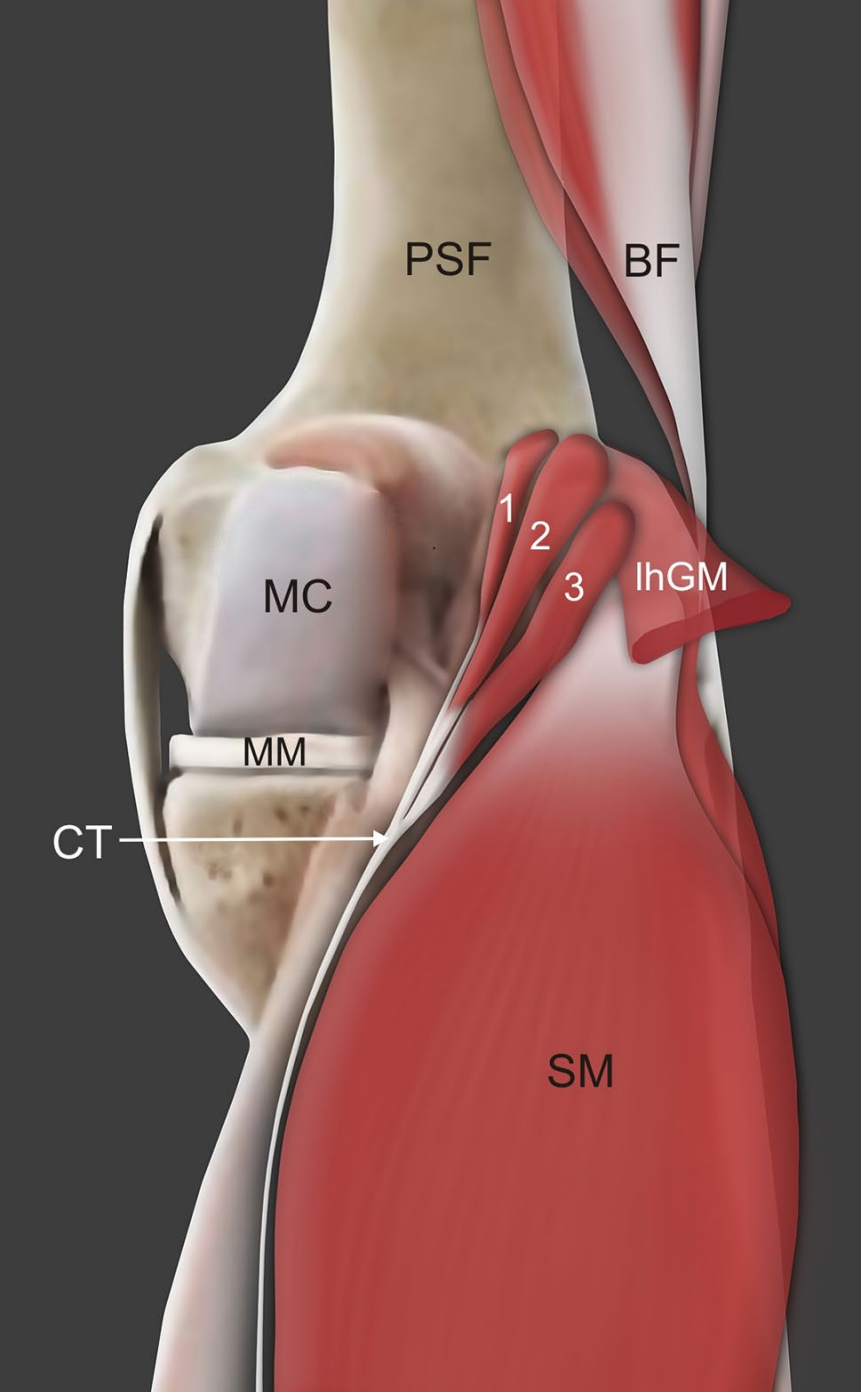
A schematic drawing of the three-headed plantaris muscle. *1* first head of the plantaris muscle, *2* s head of the plantaris muscle, *3* third head of the plantaris muscle, *TN* tibial nerve, *lhGM* lateral head of the gastrocnemius muscle, *CT* common tendon, *SM* soleus muscle, *MC* medial condyle, *PSF* posterior surface of the femur

Dissection began with removal of the skin and superficial fascia from the area of the knee and leg up to the GM. The lateral and medial heads of the proximal part of the GM were then isolated from each other. The medial head was then partially removed, and the lateral head was separated at the muscular–tendon junction, thus exposing the proximal parts of the SM and PM. The anatomical structures around the PM were then cleaned. A three-headed PM was observed. The first head (39.1 mm long) originated on the posterior femoral surface and on the medial side of the lateral femoral condyle, the second (58.72 mm long) on the lateral femoral condyle and from the lateral head of the GM, and the third (53.32 mm long) from the lateral head of the GM. The tendon of the first head was connected to the tendon of the second. Another tendon (23.22 mm) then joined the third head tendon to form a common tendon 311.23 mm long (Figs. 1, 2).

### Variations in the course of the tendon

Analysis of the course of the PM in relation to the calcaneal tendon, from its beginning at the belly of the muscle, revealed a new course variant. In this variant, the PM tendon was on the medial side of the SM. It ran medial to the GM and SM rather than in the “space” between them, eventually reaching the medial crural region.

The muscle was measured using an electronic calliper (Mitutoyo Corporation, Kawasaki-shi, Kanagawa, Japan). Each measurement was obtained twice with an accuracy of up to 0.1 mm.

## Discussion

The scientific community is very much divided by opinions about whether the PM is a residual or a developing muscle. Some authors believe because the PM is absent in 7–20% of the population, it must be a vestigial muscle [1, 7, 10, 17, 26–28]. However, it is possible that the three-headed PM described in the present report could simply be in the process of development. Unilateral and double PMs have been described previously [14, 26]. For example, Upasna and Kumar [33] describe a rare case in which the PM had a bicipital origin on both sides, the first slip arising from the lower part of the lateral supracondylar line and the second from the posterior surface of the lateral condyle of the femur. A similar case was described by Soni et al. [29]; however, unlike the previous case, no fibers merging the lateral head of the GM were observed. Kwinter et al. [14] and Rana et al. [26] both described a double PM; however, the former identified it only on the right lower limb, while the latter found it on both sides. In addition, Olewnik et al. [21] reported a double PM: the main PM originated on the lateral condyle, knee joint capsule and iliotibial band, and the accessory PM on the iliotibial band; the distal attachment showed fusion with the oblique popliteal ligament and connected with the direct arms of the semimembranosus tendon [21].

In contrast, Srimani et al. [30] described a bifurcated PM: one head (lateral) was thicker and arose from the lower part of the lateral supracondylar line deep to the lateral head of the GM, while the other (medial) was smaller and originated from the lower and medial aspect of the oblique popliteal ligament deep to the medial head of the GM. Olewnik et al. [21] also described a bifurcated PM. In this case, the lateral head of the PM originated from the lateral head of the GM, and the medial head from the knee joint capsule under the lateral head of the GM. The common tendon of the PM passed deep to both the GM and SM heads [21]. Kalniev et al. [12] observed an interesting case, an additional PM originating from the SM. Testut [32] and Le Double [4] speculated on the correlation between accessory SM and PM variability. Testut [32] thought the accessory SM as a variety of PM, but Le Double [4] refuted this view. Le Double described that additional SM can occur regardless of the morphological variability of PM [4]. Additional SM were described by several authors [5, 6, 8, 16], but none of them have already described the correlation between PM and SM.

The present case report is the first description of a three-headed PM; the first head originated on the posterior femoral surface and on the medial side of the lateral femoral condyle, the second on the lateral femoral condyle and from the lateral head of the GM, and the third from the lateral head of the GM. The three muscle bellies then united to form a common tendon and inserted on to the calcaneal tuberosity on the medial side of the calcaneal tendon.

It is unclear whether the wide variability of the PM, presenting in bifurcated, double and even three-headed forms, suggests that the muscle is just developing or forming. Although such variability indicates that an extra muscle is likely to be formed sometime in the future, the nature of this development for the fibularis tertius remains unclear [18]. From a phylogenetic perspective, it has been proposed that the PM has regressed from being a primitive flexor of the toes into a vestigial element [3]; however, the frequency of different PM variants suggests that this is not necessarily the case.

Previous studies on the morphological variability of the proximal attachment have led to the proposal of a new sixfold classification of PM origin [21]. The three-headed PM described herein perfectly matches the Type VI class, which includes all rare PM origin types.

Earlier morphometric measurements focused on muscle belly length, which ranged from 40 to 100 mm [1, 2, 7]. However, Olewnik et al. [21] measured the morphometric measurements of the muscle belly of each type of PM origin and found significant statistical differences between types.

The course of the plantaris tendon can be classified into two variants (A–B), A being the more common [22, 23]. In variant A, the tendon runs from the space between the GM and SM to the medial part of the leg; it is located on the medial side of the calcaneal tendon. In variant B, the initial course resembles that of variant A; however, upon leaving the space between the GM and SM, it turns towards the medial crural region and runs directly anterior to the calcaneal tendon [22, 23]. The present case follows a different course: the PM tendon is present on the medial side of the SM and runs medial to the GM and SM to the medial crural region rather than in the “space” between them. We, therefore, propose that the existing classification of variants of the PM tendon should be extended to include “Variant C—rare cases”. Rare tendon variants should be classed in this category and their relationship with the distal attachment checked.

## Conclusion

The plantaris muscle appears not to be a vestigial muscle but a developing one; nevertheless, knowledge of its potential morphological variability is essential for all clinicians as both its proximal and distal attachment, and the course of the PM tendon, can be implicated in a number of conditions. The classification of the plantaris muscle tendon should be extended to include “variant C”, to comprise only rare types.

## References

[1] Aragão JA, Reis FP, Guerra DR, Cabral RH (2010). The occurrence of the plantaris muscle and its muscle-tendon relationship in adult human cadavers. Int J Morphol.

[2] Chudzinski T (1882). Contributions a l’etude des variations musculaires dans les races humaines. Rev d’Anthropologie.

[3] Daselar E, Anson B (1943). The plantaris muscle: an anatomical study of 750 specimens. J Bone Jt Surg Am.

[4] Le Double A (1897). Traité des variations du système musculaire de l’homme et de leur signification au point de vue de l’anthropologie zoologique.

[5] Featherstone T (1995). MRI diagnosis of accessory soleus muscle strain. Br J Sports Med.

[6] Flower JM (1931). Note on an accessory soleus muscle. J Anat.

[7] Freeman AJ, Jacobson NA, Fogg QA (2008). Anatomical variations of the plantaris muscle and a potential role in patellofemoral pain syndrome. Clin Anat.

[8] Garnier C, Villemin F (1909). Muscles soléaires accessoires chez l’homme. Bibliog Anat Par Nancy.

[9] Gonera B, Kurtys K, Karauda P, Olewnik PM (2020). Possible effect of morphological variations of plantaris muscle tendon on harvesting at reconstruction surgery-case report. Surg Radiol Anat.

[10] Harvey FJ, Chu G, Harvey PM (1983). Surgical availability of the plantaris tendon. J Hand Surg Am.

[11] Joshi MM, Joshi SD, Joshi SS (2014). Morphological variations of muscle plantaris: anatomical and clinical insight. Int J Anat Res.

[12] Kalniev MA, Krastev DS, Krastev NS, Apostolov AM, Mileva MM (2014). An unusual variation of an additional plantaris originating from the soleus—a histological examination. Int J Adv Res Biol Sci.

[13] Kurtys K, Gonera B, Olewnik KP, Polguj M (2020). A highly complex variant of the plantaris tendon insertion and its potential clinical relevance. Anat Sci Int.

[14] Kwinter D, Lagrew J, Lawrence C (2010). Unilateral double plantaris muscle: a rare anatomical variation. Int J Morphol.

[15] Moore K, Dalley A (2006). Lower limb clinically oriented anatomy.

[16] Motto S, Holloway G (1996). The accessory soleus muscle. Br J Sports Med.

[17] Nayak SR, Krishnamurthy A, Ramanathan L, Ranade AV, Prabhu LV, Jiji PJ, Rai R, Chettiar GK, Potu BK (2010). Anatomy of plantaris muscle: a study in adult Indians. Clin Ter.

[18] Olewnik Ł (2019). Fibularis tertius: anatomical study and review of the literature. Clin Anat.

[19] Olewnik Ł, Gonera B, Podgórski M, Polguj M, Jezierski H, Topol M (2019). A proposal for a new classification of pes anserinus morphology. Knee Surg Sport Traumatol Arthrosc.

[20] Olewnik Ł, Karauda P, Gonera B, Kurtys K, Haładaj R, Tubbs RS, Paulsen F, Ramón Sanudo J, Polguj M (2020). Intramuscular innervation of plantaris muscle evaluated using a modified Sihler’s staining protocol—proposal for a new classification. Ann Anat Anat Anzeiger.

[21] Olewnik Ł, Kurtys K, Gonera B, Podgórski M, Sibiński M, Polguj M (2020). Proposal for a new classification of plantaris muscle origin and its potential effect on the knee joint. Ann Anat Anat Anzeiger.

[22] Olewnik L, Wysiadecki G, Podgórski M, Polguj M, Topol M (2018). The plantaris muscle tendon and its relationship with the Achilles tendinopathy. Biomed Res Int.

[23] Olewnik Ł, Wysiadecki G, Polguj M, Topol M (2017). Anatomic study suggests that the morphology of the plantaris tendon may be related to Achilles tendonitis. Surg Radiol Anat.

[24] Olewnik PM, Polguj M, Topol M (2018). The plantaris muscle—rare relations to the neurovascular bundle in the popliteal fossa. Folia Morphol.

[25] Olewnik WG, Polguj M, Topol M (2017). The report on the co-occurrence of two different rare anatomic variations of the plantaris muscle tendon on both sides of an individual. Folia Morphol.

[26] Rana KK, Das S, Verma R (2006). Double plantaris muscle: a cadaveric study with clinical importance. Int J Morphol.

[27] Rohilla S, Jain N, Yadav R (2013). Plantaris rupture: why is it important?. Case Rep.

[28] Simpson SL, Hertzog MS, Barja RH (1991). The plantaris tendon graft: an ultrasound study. J Hand Surg Am.

[29] Soni S, Saxena A, Ghulyani T, Rani-Das A (2014). A biceps plantaris in the popliteal region—case report. Eur J Anat.

[30] Srimani P, Meyur R, De Bose A, Kundu B, Sadhu A (2014). Unilateral variation of plantaris muscle: a case report. J Evol Med Dent Sci.

[31] Van Sterkenburg MN, Kerkhoffs GMMJ, Kleipool RP, Niek Van Dijk C (2011). The plantaris tendon and a potential role in mid-portion Achilles tendinopathy: an observational anatomical study. J Anat.

[32] Testut L (1884). Les anomalies musculaires chez l’homme: expliquées par l’anatomie comparée leur importance en anthropologie.

[33] Upasna KA (2011). Bicipital origin of plantaris muscle—a case report. Int J Anat Var.

